# A transition point: Assistance magnitude is a critical parameter when providing assistance during walking with an energy-removing exoskeleton or biomechanical energy harvester

**DOI:** 10.1371/journal.pone.0289811

**Published:** 2023-08-10

**Authors:** Michael Shepertycky, Yan-Fei Liu, Qingguo Li

**Affiliations:** 1 Department of Mechanical and Materials Engineering, Queen’s University, Kingston, Canada; 2 Department of Electrical and Computer Engineering, Queen’s University, Kingston, Canada; New Jersey Institute of Technology, UNITED STATES

## Abstract

Researchers and engineers have developed exoskeletons capable of reducing the energetic cost of walking by decreasing the force their users’ muscles are required to produce while contracting. The metabolic effect of assisting concentric and isometric muscle contractions depends, in part, on assistance magnitude. We conducted human treadmill experiments to explore the effects of assistance magnitude on the biomechanics and energetics of walking with an energy-removing exoskeleton designed to assist eccentric muscle contractions. Our results demonstrate that the assistance magnitude of an energy-removing device significantly affects the energetics, muscle activity, and biomechanics of walking. Under the moderate assistance magnitude condition, our device reduced the metabolic cost of walking below that of normal walking by 3.4% while simultaneously producing 0.29 W of electricity. This reduction in the energetic cost of walking was also associated with an 8.9% decrease in hamstring activity. Furthermore, we determined that there is an assistance magnitude threshold that, when crossed, results in the device transitioning from assisting to hindering its user. This transition is marked by significant increases in muscle activity and the metabolic cost of walking. These results could aid in the future design of exoskeletons and biomechanical energy harvesters, as well as adaptive control systems, that identify user-specific control parameters associated with minimum energy expenditure.

## Introduction

Walking, our primary form of locomotion, is metabolically expensive. Humans expend more energy while walking than during any other activity of daily living [1,2]. Active force production by muscles is the main source of walking’s high energetic demand [3]. To reduce fatigue and injury risk, researchers have developed lower-limb exoskeletons that can decrease the high energetic cost of walking by reducing the force that muscles naturally need to produce. Exoskeletons that reduce the metabolic cost of walking can also promote prolonged walking, which is associated with improved physical and mental health and quality of life [4].

These exoskeletons have three main modes of operation: injecting, transferring, and removing energy [5]. Active exoskeletons inject energy into the human–machine system to assist concentrically contracting muscles. For example, an active hip exoskeleton developed by Kim et al. uses electrical power stored in a battery to power the device’s electric motors and control system [6]. The electric motor works in conjunction with a Bowden cable to assist the gluteus maximus in producing a hip extension moment during the loading response and mid stance phases of gait. These devices, in part, replace muscles with actuators that are commonly powered by batteries or compressed gas. One limitation of these devices is that their operational lifespans are limited by the energy capacity of their energy sources.

Passive exoskeletons assist isometrically contracting muscles by capturing and transferring energy from one phase of gait to another, decreasing the amount of force that muscle–tendon units (e.g., the soleus-achilles tendon unit) must hold. For example, Collins et al. developed a passive ankle exoskeleton that consists of a clutch and spring system situated parallel to the user’s soleus and gastrocnemius muscle-tendon unit. This device collects energy during the mid and terminal stance phases and returns it during the pre-swing phase [7]. Although the operational life of these devices does not depend on the energy capacity of an energy source, they are not without limitations. One limitation is that, because these devices use passive mechanisms to store and return harvested energy, they cannot use active control systems to identify users’ specific needs and may not provide optimal assistance.

Energy-removing exoskeletons assist eccentrically contracting muscles by removing kinetic energy that is naturally dissipated by muscles. For example, a knee-based exoskeleton developed by Shepertycky et al. used a generator and cable system to assist the hamstring muscles in producing a knee flexion moment by removing energy during the terminal swing phase [8]. One potential advantage of energy-removing exoskeletons is that their control systems can be self-powering because they can convert the removed energy into electricity. Energy-removing exoskeletons are similar to biomechanical energy harvesters in that they both remove energy from their users and convert it into electricity.

The metabolic effect of assisting concentric and isometric muscle contractions depends, in part, on assistance magnitude [7,9–12]. Assistance magnitude is the amount of mechanical force, moment, or power the device applies to the user; it has been quantified using many different measures, including the percentage of the peak biological joint moment [11] and the average mechanical power applied by the exoskeleton [12]. The metabolic cost of walking while assisting concentric and isometric muscle contractions exhibits a quadratic relationship with the device’s assistance magnitude, with a particular magnitude associated with the greatest metabolic cost reduction [7,10]. For example, Collins et al. [7] identified a quadratic relationship between the metabolic cost of walking with their passive ankle exoskeleton and spring stiffness, a feature associated with device assistance magnitude (*R*^*2*^ = 0.91, *p* = 0.029). In their study, the lowest metabolic cost of level walking was associated with the device using a moderately stiff spring (i.e., 180 Nm·rad^-1^); the use of springs with greater or lesser stiffness was associated with higher metabolic costs. Similarly, Kang et al. [10] identified a quadratic relationship between the metabolic cost of walking with their active hip exoskeleton and the assistance magnitude (*R*^*2*^ = 0.869, *p* < 0.01). The authors used the identified quadratic model to estimate the greatest possible metabolic cost reduction and the associated assistance magnitude. They estimated that an assistance magnitude equivalent to 20% of the peak biological hip moment was associated with the maximal metabolic cost reduction of 6% compared to that of walking with the unpowered exoskeleton.

Researchers have designed adaptive control systems that incorporate search algorithms that tune control parameters (e.g., assistance magnitude, peak timing, duration), based on real-time physiological measurements to provide the user with greater levels of assistance. These adaptive control systems have identified user-specific assistance profiles that have resulted in greater reductions in metabolic cost than those of non-adaptive systems [13–15]. In addition to greater metabolic reductions, these adaptive control systems decrease user-specific profile identification times without relying on a researcher’s intuition or repeated trial-and-error experiments [13–15].

Adaptive control systems rely on a reasonable understanding of the metabolic effects of altering given control parameters. Without an understanding of the parameter–metabolic cost landscape, an algorithm may be set to search too large a parameter space and may fail to find an appropriate value. Additionally, without a thorough understanding of the parameter space, the algorithm may search an area in which the global minimum does not exist, causing it to identify a local minimum or even get stuck searching along a plateau [15]. For example, an algorithm designed by Zhang et al. [14] that optimized parameters of an active tethered ankle exoskeleton’s loading profile required 64 min to identify an optimal assistance profile for nine of 11 participants. However, the control system required more time to identify an optimal assistance profile for the remaining two participants (128 and 208 min) because it got trapped in a local minimum and was reset. Notably, because the authors understood the parameter–metabolic cost landscape from previous experiments using the same device [14,16], they recognized that the algorithm had become trapped in a local minimum and reset it accordingly.

Unfortunately, the assistance magnitude–metabolic cost landscape associated with assisting eccentric contractions using an energy-removing device (i.e., an energy-removing exoskeleton or biomechanical energy harvester) is largely unknown because although energy-removing devices have previously produced electricity efficiently, their use has negatively impacted their users’ performance (i.e., by increasing the metabolic cost) [17–24]. Therefore, previous studies that have evaluated assistance or harvesting magnitudes only examined a range of magnitudes in which the devices did not provide user assistance [21,24,25] and an adaptive control system would not operate.

Shepertycky et al. demonstrated for the first time that the metabolic cost of walking could be reduced by solely removing energy [8]. Their device applied a moment about the user’s knee that mimicked the contribution of the user’s muscles during the terminal swing phase of gait, resulting in a 2.5% reduction in the metabolic cost of walking and an 11.2% decrease in hamstring activity while producing 0.25 W of electricity [8]. Additionally, Shepertycky et al. determined that, similar to active exoskeletons [13,26–28], the timing of assistance was critical in reducing the metabolic cost of walking. However, the effect of assistance magnitude on the metabolic cost of walking while assisting eccentric contractions remains unknown.

Here, we explore the energetic and biomechanical effects of an energy-removing device that applies differing assistance magnitudes to elucidate the effects of assisting eccentric muscle contractions. Because assisting eccentric contractions should be similar to assisting isometric and concentric contractions, we hypothesized there would be a quadratic relationship between our device’s assistance magnitude and the metabolic cost of walking.

## Materials and methods

### Energy-removing exoskeleton

The energy-removing exoskeleton we evaluated here is the same device described in [8,29]. A detailed description of the exoskeleton’s control system and the muscle-centric assistance profile we utilized are described in [30] and the supplementary materials of [8]. A brief description of the exoskeleton’s operation is included here for completeness.

**Fig 1:**
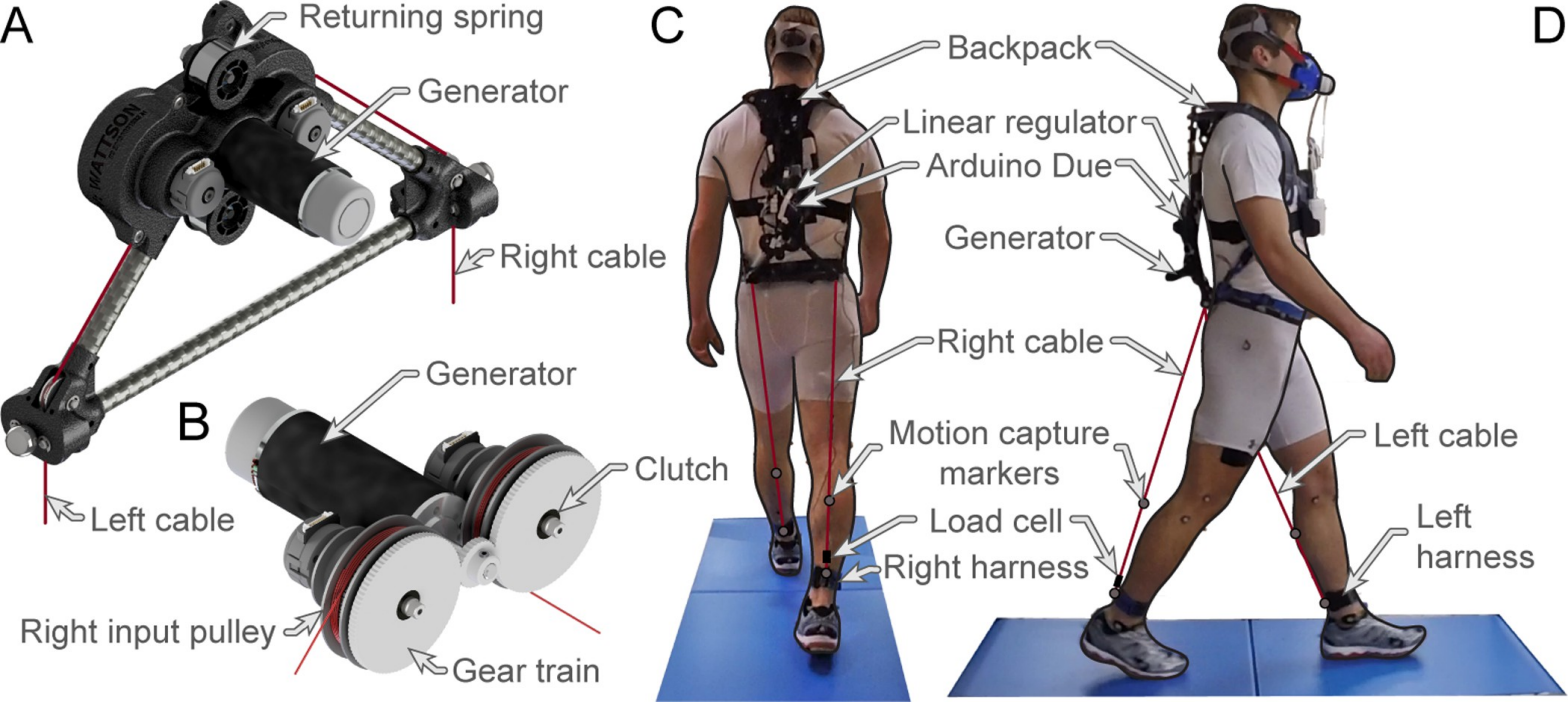
Energy-removing exoskeleton. Isometric views of the energy-removing exoskeleton (A) and its internal components (B). A back (C) and side (D) view of a participant walking with the exoskeleton and experimental equipment on the force-sensing treadmill.

The backpack-mounted energy-removing exoskeleton assists the eccentrically contracting hamstrings by applying a knee flexion moment during the terminal swing of gait via two cables (one per leg). These cables extend down from the device (Fig 1A and 1B) and attach to the user’s lower shanks, just above the ankle, via shank harnesses (Fig 1C and 1D). The opposite end of each cable is connected to its respective input pulley. As the user’s leg swings forward during the swing period, the cable is unspooled from the input pulley, driving the gear train. The device’s gear train, in combination with its unidirectional roller clutches (S99NH3MURC0612, SDP/SI, USA), integrates and amplifies the input motion before engaging the generator (EC-4 pole 305015, Maxon Motor, Switzerland) [29]. During the stance period, the clutches decouple the input pulley from the gear train, allowing the cable to be spooled back onto the input pulley by a returning spring. Additionally, this decoupling enables the device to apply mechanical load only during the swing period [8]. The electromagnetic force produced by the generator and applied to the user by the input cables is controlled using a custom-designed linear regulator and an Arduino-based control system [30,31]. This control system uses a hybrid approach consisting of an open-loop control scheme for predicting and applying the desired load and a closed-loop feedback control scheme for high-level regulation of the applied angular impulse. The loading profile applied by the control system was mapped as a function of gait cycle (i.e., % gait cycle).

### Participants

Twelve healthy adult males (age = 28.6 ± 2.5 years, mass = 77.4 ± 3.8 kg, height = 1.77 ± 0.02 m, mean ± S.E.M.) participated in this study after providing informed written consent. Our sample size was based on that of previous studies that employed similar experimental methodologies [7,8,21]. No participants reported having known or apparent injuries that could affect their gait, and we did not exclude any data from the analyses. The study protocol was approved by the General Research Ethics Board of Queen’s University (TRAQ: 6006569).

### Testing protocol

The human walking experiments consisted of participants performing the following five random-order treadmill walking activities: 1) normal walking, in which the participant walked without the exoskeleton; 2) weighted walking, in which the participant walked with the exoskeleton but with the input cables disconnected from the shank harnesses; and 3) walking under the three assistance conditions, in which the participants walked with the exoskeleton applying the muscle-centric loading profile with an assistance magnitude of 10%, 15%, or 20% of the muscles’ estimated contribution to the negative angular impulse of the knee during the swing phase. The muscle-centric loading profile was developed by Shepertycky et al. [8]. This profile was designed to resemble the muscles’ contribution to the net knee moment during the terminal swing phase of gait. Shepertycky et al. derived this profile by subtracting the estimated contribution of passive elements (e.g., ligaments) from the net knee moment profile. They estimated the contribution of the passive elements based on the results of a study conducted by Whittington et al. [32]. The weighted walking activity was considered the 0% assistance condition. Each activity lasted for 10 min and was followed by a 5-min rest period. Each activity was completed using a level split-belt force-sensing treadmill at a speed of 1.25 m·s^-1^ (AMTI Inc., MA, USA). We collected data during a two-min analysis window [7,33,34], starting at the 7.5 min mark of each trial. We chose this analysis window to allow the participants enough time to adapt to the walking conditions while preventing potential end effects.

Before data collection, participants performed two acclimation activities. The first acclimation activity occurred one to three days before data collection. The second acclimation activity occurred immediately before the first of the five walking activities on the day of data collection. The first acclimation activity consisted of the participant walking on a single-belt treadmill (Model #1930, IRONMAN Fitness, Florida, USA) with the exoskeleton applying the muscle-centric loading profile at a randomly selected assistance magnitude (e.g., 10%, 15%, or 20%) for 20 min. This activity allowed the participants to gain experience walking with the device. During the second acclimation activity, each participant walked on the split-belt force-sensing treadmill for 10 min without the exoskeleton (similar to the normal walking activity). This activity allowed the participants to gain experience walking on the split-belt treadmill while wearing the testing equipment (e.g., the indirect calorimetry mask) [35]. We chose walking without the exoskeleton for this second acclimation activity to ensure that the participants did not receive disproportionate training in one of the exoskeleton conditions on the day of testing. Before the second acclimation activity, we determined each participant’s basal metabolic rate during a 10-min quiet standing trial.

### Assistance levels

Assistance magnitude was defined as the magnitude of the angular impulse applied by the device about the user’s knee during the swing phase—as in our previous study [8]. We identified the angular impulse by calculating the integral of the moment applied by the exoskeleton about the user’s knee in the sagittal plane with respect to time. We selected the three assistance levels (low: 10%, moderate: 15%, and high: 20%) based on our experience developing and evaluating both the energy-removing exoskeleton [8] and the lower-limb-driven energy harvester [21,36]. For instance, we chose the 20% assistance magnitude as the upper assistance magnitude because observations made during the control system development [30] and participant feedback suggested that this assistance level might hinder normal walking. Furthermore, we could not evaluate an assistance level between 0% and 10% because it would have been within the exoskeleton’s minimum capabilities; more specifically, the minimum force required to drive the exoskeleton’s mechanical system would cause the applied impulse to be higher than the desired impulse or assistance magnitude [8,30].

### Data collection

We examined the kinetics, kinematics, and muscle activity from the participant’s right leg—the left leg was assumed to behave symmetrically [6,8,34]. We used an AMTI Force-Sensing Tandem Treadmill (AMTI Inc., MA, USA; sample rate: 1,000 Hz) and a seven-camera motion capture system (Oqus, Qualisys, Sweden; sample rate: 100 Hz) to measure ground reaction forces and kinematics, respectively. We measured the whole-body oxygen consumption and carbon dioxide production using a K4B2 indirect calorimetry system (COSMED, Italy). We used a wireless surface electromyography (sEMG) system (Trigno, DELSYS, MA, USA; sample rate: 2,000 Hz) to measure the activity of three quadriceps muscles (vastus lateralis, vastus medialis, and rectus femoris) and two hamstring muscles (semitendinosus, biceps femoris). The surface electrode placements were based on Surface ElectroMyoGraphy for the Non-Invasive Assessment of Muscles (SENIAM) project recommendations [37]. We acquired the voltage and current produced by the exoskeleton and the input cable force using a wireless analog adaptor (Trigno ± 5V Adaptor, DELSYS, MA, USA; sample rate: 2,000 Hz). We measured the current and voltage generated by the exoskeleton across the current-sense resistor (0.05 Ω, PF2205-0R05J1, RIEDON, CA, USA) and the electrical load, respectively [21,36]. We used a single-axis load cell (ZNLS-10KG, Anhui Connaught Sensor Co., Ltd., China) mounted between the input cable and the right shank harness to measure the input cable force.

### Data analysis

We segmented the kinematic, kinetic, sEMG, and device performance data from each trial into gait cycles, defined as the period between subsequent heel strikes of the right leg. We excluded a gait cycle from the analysis if both of the participant’s feet were on the same force plate at the instance of ground contact. We examined the first 20 consecutive gait cycles within the analysis window that met the inclusion criterion based on previous studies [8,9]. This approach ensures consistency across participants and minimizes the effects of outliers. We calculated the average gait cycle period and stride length for each participant and activity. A stride length was calculated as the sum of sequential right and left leg step lengths, whereas step length was calculated as the fore–aft distance between the two heel markers at the instance of ground contact. We calculated the average kinematics, kinetics, muscle activity, and device performance measures over the 20 gait cycles and 12 participants.

We used an inverse dynamics approach to determine the ankle, knee, and hip joint kinematics and kinetics (i.e., joint angles, net joint moments, and powers) [38]. We normalized the joint moments and powers to each participant’s body mass. We calculated the muscles’ estimated contributions to the net joint moments as was previously done in [8]; this method consisted of subtracting both the device’s contribution [7,39] and the contribution of the passive elements, estimated based on results reported by Whittington et al. [32], from the net joint moment. We determined the device’s contribution by calculating the cross-product of the cable force and the moment arm the cable made with the knee joint center. We used the coordinates of reflective markers placed on the cable attachment point of the shank harness and the cable insertion point on the exoskeleton to determine the moment arm and the cable force vectors [8,21]. We estimated the impulse applied by a given element (e.g., the device) by taking the time-integral of the moment applied by the element over the period of interest.

We determined the metabolic rate of each walking activity using a standard equation [40] based on the carbon dioxide production and average oxygen consumption calculated during the two-min analysis window. We then normalized the metabolic energy demand of each of the five walking activities by first subtracting the activity’s basal metabolic rate from the gross metabolic rate of the activity [7,32,33] and dividing the result by the participant’s body mass. We identified the basal metabolic rate from the quiet standing trial.

We processed the sEMG data as follows. We first high-pass filtered the sEMG signals measured from the five right leg muscles at 20 Hz using a four-order zero-phase shift Butterworth filter. We then full-wave rectified the resulting signals. Subsequently, we created muscle activity linear envelopes by low-pass filtering the full-wave rectified signals at 10 Hz using a four-order zero-phase shift Butterworth filter [7,8,39]. Next, we normalized each muscle’s activity to the peak value observed during normal walking. As previously done [7,8], we summed the normalized muscle signals within muscle groups to simplify the analysis and interpretation of muscle activity. We took the sum of the normalized semitendinosus and biceps femoris signals to form the hamstring muscle group and sum of the the vastus lateralis, vastus medialis, and rectus femoris signals to form the quadriceps muscle group. We normalized each muscle group’s activity to the peak value observed during normal walking. We calculated each muscle group’s average activity as the time integral of the muscle group’s activity divided by the period of the gait cycle. The average muscle activities for initial contact to terminal stance (0–31% gait cycle), terminal stance to the end of pre-swing (31–62% gait cycle), initial swing (62–75% gait cycle), and mid swing to the end of terminal swing (75–100% gait cycle) were calculated as the time integral over the period, divided by the gait cycle period.

We calculated the average mechanical power removed by the exoskeleton as the time integral of the instantaneous mechanical power divided by the period of the gait cycle, multiplied by two to account for the mechanical power removed from the left leg. We calculated the instantaneous mechanical power removed by the exoskeleton by multiplying the input cable force by the input cable velocity. We determined cable velocity by taking the time derivative of the input cable length, with the cable length being the distance between the motion capture markers placed on the cable insertion point on the exoskeleton and the cable attachment point on the shank harness [8,21]. We calculated the average electrical power by dividing the time integral of the instantaneous electrical power by the gait cycle period. We determined the instantaneous electrical power by multiplying the voltage measured across the load by the current produced. The electrical power was not multiple by two because the device’s gear train integrated the motion captured from both lower limbs before it engaged the single generator; thus, we measured the electrical power produced from both legs. We determined the device’s power production efficiency by dividing the electrical power produced by the mechanical power input.

Furthermore, we calculated two energy harvesting performance measures—the Cost of Harvest (COH) [20] and the Total Cost of Harvest (TCOH) [21]—for each exoskeleton condition. We calculate the COH by dividing the metabolic difference between the exoskeleton and weighted walking conditions by the electrical power produced [20]. Additionally, we calculated the TCOH by dividing the metabolic difference between the exoskeleton and normal walking conditions by the electrical power produced [21].

### Statistics

We analyzed the effects of the walking conditions (i.e., normal, weighted, low assistance, moderate assistance, and high assistance) on each user’s metabolic rate using a repeated-measures ANOVA. We applied a Greenhouse-Geisser correction wherever Mauchly’s test of sphericity was significant [6]. If the omnibus ANOVA was significant, we conducted *post hoc* comparisons of conditions using the Šidák correction for multiple comparisons. We used this same procedure to compare the effect of assistance magnitude on additional outcomes, namely the joint kinematics (e.g., peak knee flexion angle), kinetics (e.g., peak net knee moment), muscle activity (e.g., average hamstring activity), and device performance measures (e.g., electrical power produced). We compared the applied assistance magnitudes to the desired magnitudes (i.e., 10, 15, and 20%) using one-sample t-tests. We conducted a least-squares regression analysis to fit a 2nd-order polynomial (quadratic) function relating the metabolic rate of the three assistance conditions and weighted walking to the assistance magnitude.

We assessed normality using the Shapiro-Wilk test; however, we did not perform corrections for violations of the assumption of normality because ANOVAs and t-tests are robust to non-normality, particularly when sample sizes are equal [41–43]. We have noted all normality assumption violations in the S1 Data. We performed all statistical analyses using SPSS version 28 (IBM Corporation, USA) with the criterion for statistical significance set at α = 0.05. All data are presented as the means ± S.E.M., and the results are compiled in the S1 and S2 Data.

## Results

### Metabolic and muscle activity

**Fig 2:**
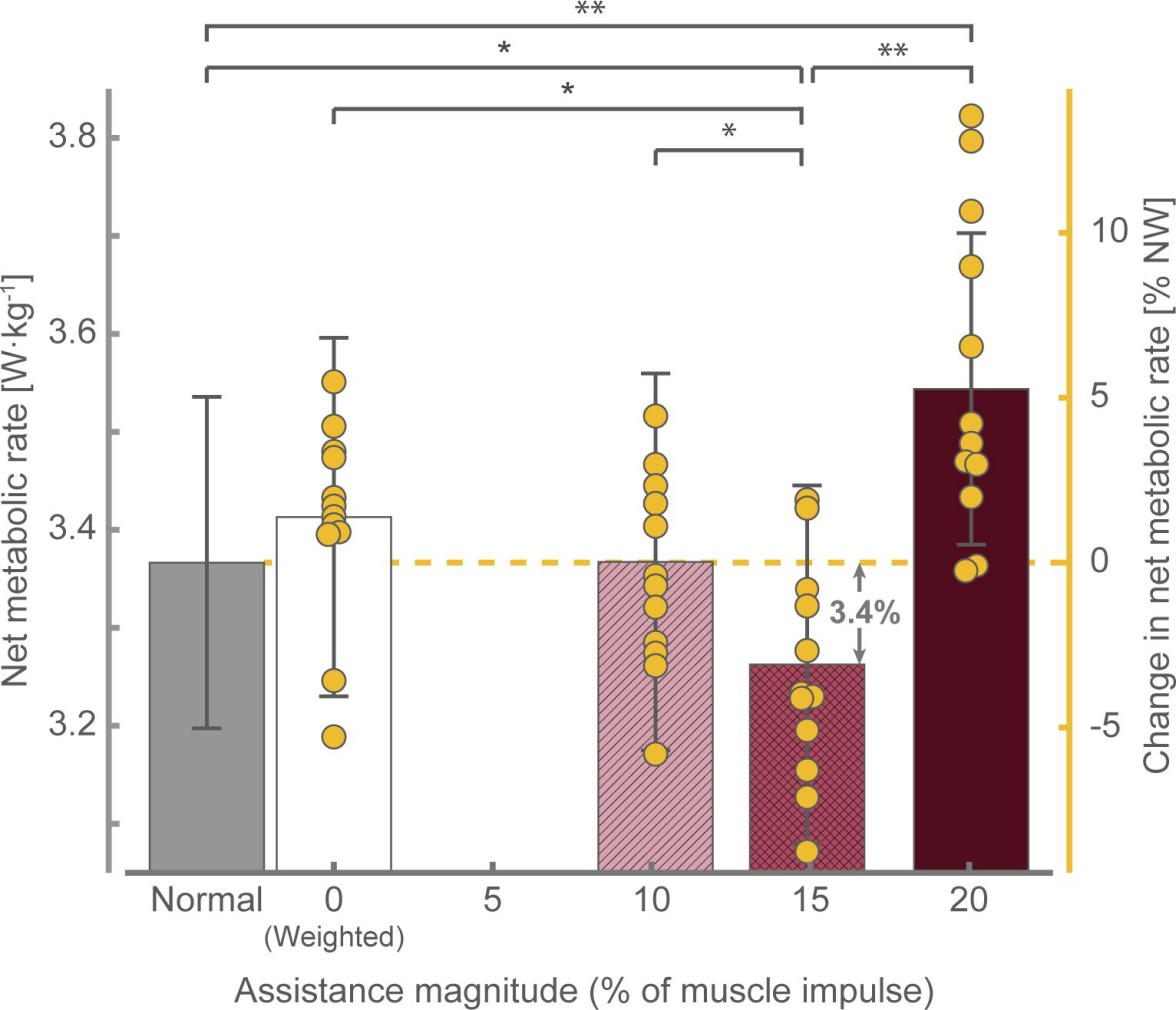
Average net metabolic rate across walking conditions. The left vertical axis represents the net metabolic rate (means and error bars), whereas the right vertical axis represents the metabolic rate as a percent change from that of NW (means and scatter). *p < 0.05, **p < 0.01. Data presented as mean ± S.E.M. NW: Normal walking.

Examination of the metabolic results revealed that the assistance magnitude significantly affected the metabolic cost of walking (*F*_*(4*,*44)*_ = 14.032, *p* < 0.001, *ηp*^*2*^ = 0.561; Fig 2). Our Šidák *post hoc* analysis revealed that the moderate assistance condition was associated with a statistically significant reduction in metabolic cost of 3.4 ± 1.0% compared to that of normal walking (mean diff.: 0.104 ± 0.029 W·kg^-1^, *p* = 0.045). Additionally, the moderate assistance condition was associated with a statistically significant reduction compared to that of the weighted (-4.5 ± 1.1%, *p* = 0.013), low assistance magnitude (-3.1 ± 0.7%, *p* = 0.025), and high assistance magnitude (-9.6 ± 2.1%, *p* = 0.003) conditions. Furthermore, the high assistance condition resulted in a statistically significant increase in metabolic cost relative to that of normal walking (5.7 ± 1.4%, *p* = 0.007). Simply wearing the exoskeleton (i.e., weighted walking or 0% assistance condition) did not significantly alter the metabolic cost of walking (mean diff. from normal walking: 0.046 ± 0.029 W·kg^-1^, *p* = 0.781). While not statistically significant, an increase in metabolic cost related to carrying the weight of the device was similar to that theoretically estimated using results reported by Browning et al. (0.059 W·kg^-1^) [44]. Further examination of the metabolic results revealed that there was no statistically significant quadratic relationship between the assistance magnitude and the change in metabolic cost from normal walking (*R*^*2*^ = 0.018; *F*_*(2*,*45)*_ = 401, *p* = 0.672).

To identify possible means through which the device affected the metabolic cost of walking, we analyzed sEMG data for the hamstring and quadriceps muscle groups. Because force production by muscles is the primary consumer of metabolic energy during walking [3], we expected to observe a decrease in muscle activation associated with the reduction in metabolic cost associated with the moderate assistance condition. Additionally, because improper energy extraction could increase the muscle activity required to balance the forces applied by the exoskeleton, we expected the increased metabolic cost of walking observed with the high assistance magnitude condition to be associated with increased muscle activity.

**Fig 3:**
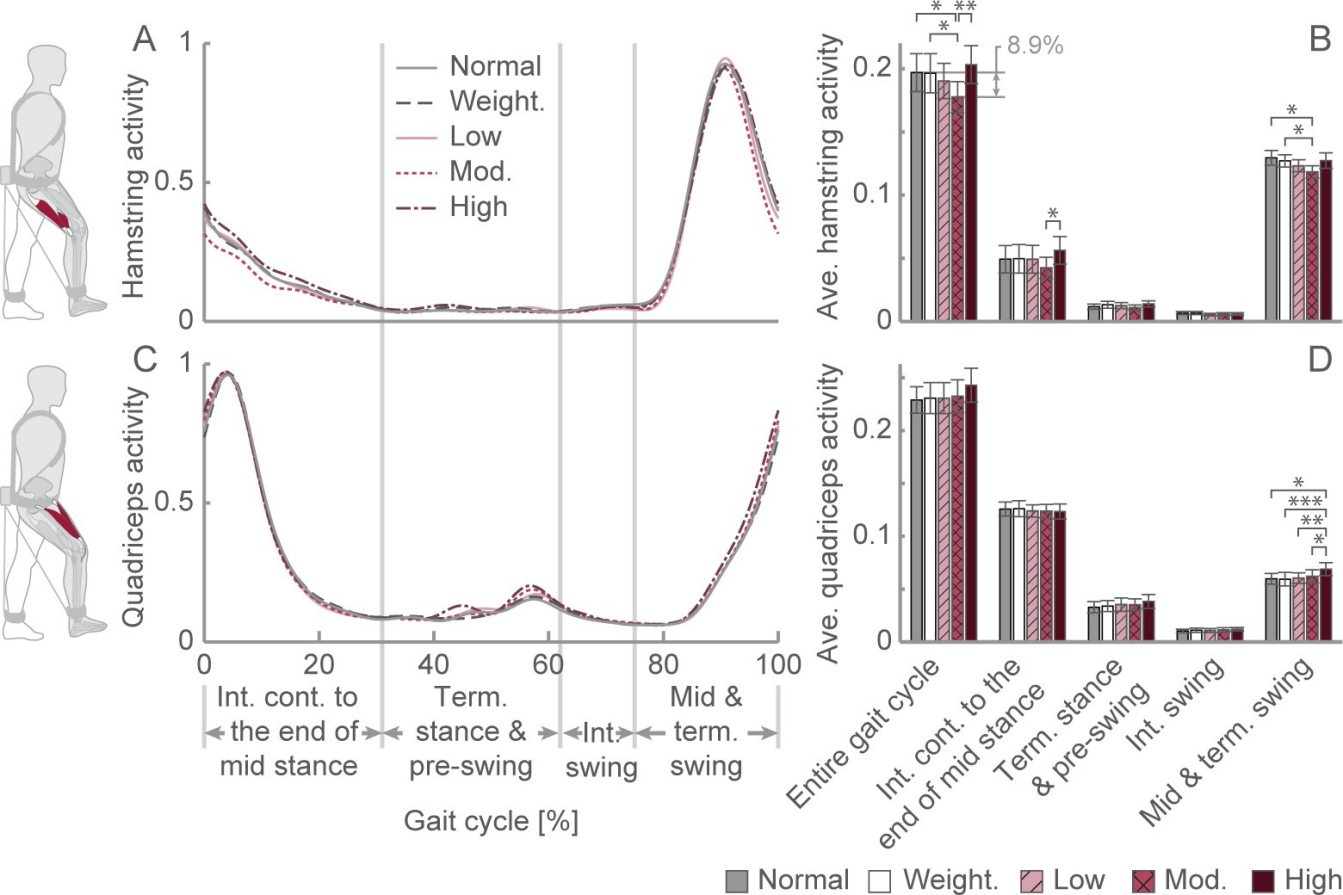
Hamstring and quadriceps activity. (A) Average hamstring activity profile over a gait cycle. (B) Average hamstring activity for a gait cycle. (C) Average quadriceps activity profile over a gait cycle. (D) Average quadriceps activity for a gait cycle. *p < 0.05, **p < 0.01, ***p < 0.001. Data presented as the mean ± S.E.M. Ave.: Average; Cont.: Contact; Int.: Initial; Mod.: Moderate; Term.: Terminal; Weight.: Weighted.

Our examination of the sEMG results identified that assistance magnitude significantly affected hamstring activity (0–100% gait cycle**,** *F*_(*4*,*44*)_ = 7.580, *p* < 0.001, *ηp*^2^ = 0.408; Fig 3A and 3B). The Šidák *post hoc* analysis revealed that the hamstring activity over the entire gait cycle of the moderate assistance condition was significantly lower than that of the normal (8.9 ± 2.3%, *p* = 0.041), weighted (8.6 ± 2.1%, *p* = 0.035), and high assistance (11.8± 2.4%, *p* = 0.004) conditions. This decrease relative to normal and weighted walking primarily occurred during mid and terminal swing (75–100% gait cycle; *F*_*(2*.*551*,*28*.*060)*_ = 5.020, *p* = 0.009, *ηp*^2^ = 0.313; Šidák *post hoc* analysis normal: *p* = 0.049, weighted: 0.021), while the decrease relative to the high assistance condition primarily occurred during the beginning of stance (0–31% gait cycle; *F*_*(4*,*44)*_ = 4.609, *p* = 0.003, *ηp*^2^ = 0.295; Šidák *post hoc* analysis: *p* = 0.013). This result provides further evidence that the reduction in metabolic cost associated with our energy-removing exoskeleton is related to the reduction in hamstring activity.

Although assistance magnitude did not significantly affect overall quadricep activity (0–100% gait cycle; *F*_(*4*,*44*)_ = 1.794, *p* = 0.147, *ηp*^2^ = 0.140; Fig 3C and 3D), it did significantly affect quadricep activity during mid and terminal swing (75–100% gait cycle; *F*_(*4*,*44*)_ = 8.226, *p* < 0.001, *ηp*^2^ = 0.428). The *post hoc* analysis identified that quadricep activity during late swing (75–100% gait cycle) of the high assistance condition was significantly greater than that of the normal (*p* = 0.044), weighted (*p* < 0.001), low assistance (*p* = 0.003), and moderate assistance (*p* = 0.030) conditions. Data for individual hamstring and quadriceps muscles are shown in S1 and S2 Figs.

The biarticular nature of the rectus femoris—crossing both the knee and hip—results in the muscle contributing to knee extension and hip flexion. Therefore, we analyzed the rectus femoris activity independent of the quadriceps group to examine whether the exoskeleton affected the user’s hip. Our examination of the rectus femoris activity did not reveal any significant differences between the walking conditions during the entire gait cycle (0–100% gait cycle; *F*_*(2*.*125*,*23*.*374)*_ = 2.184, *p* = 0.133, *ηp*^2^ = 0.166; S2C and S2D Fig) or during any of the four gait divisions**.**

### Kinematics and kinetics

**Fig 4:**
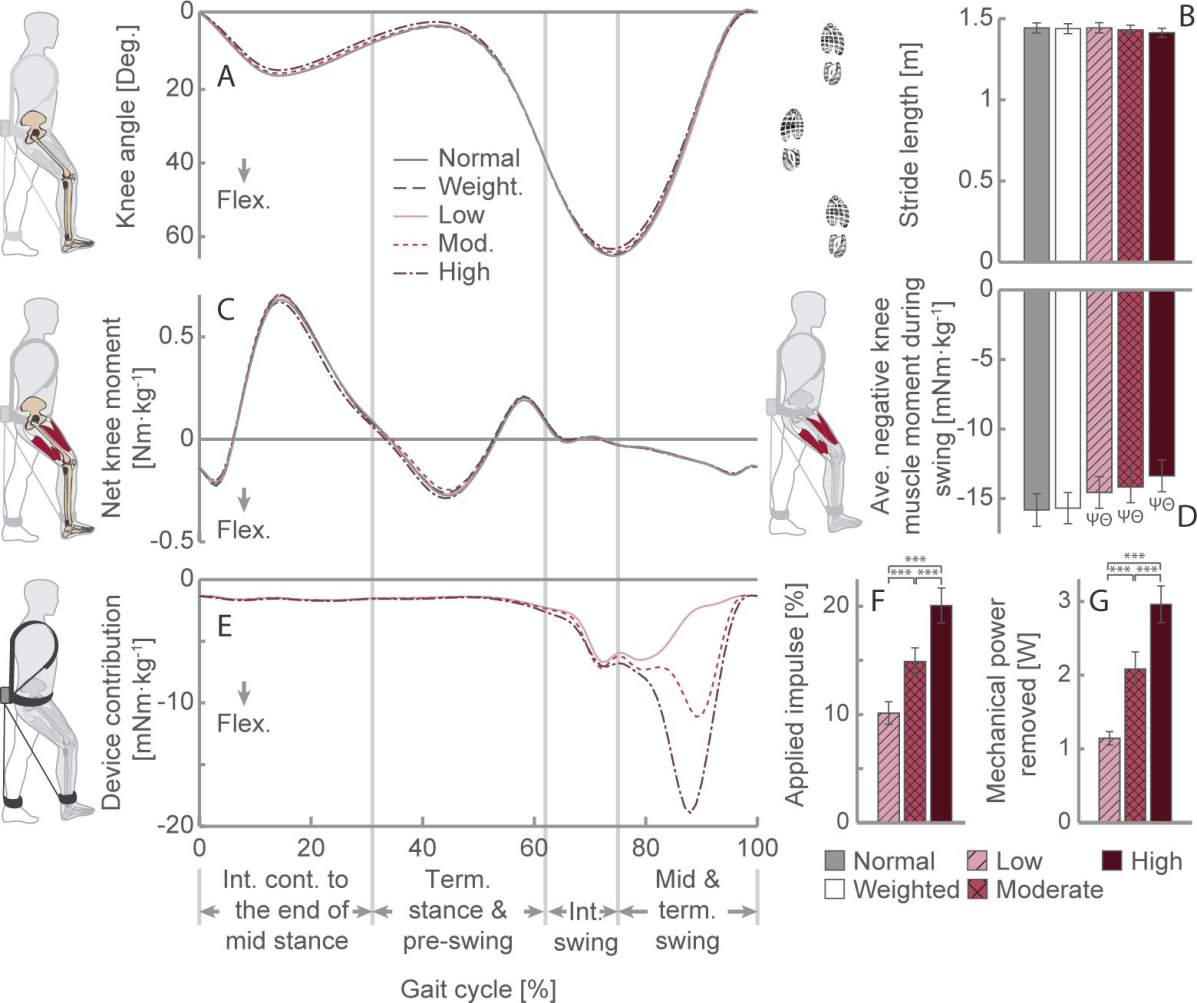
Right leg sagittal plane knee kinematics and kinetics, averaged across participants. (A) Knee joint angles over a gait cycle for five walking activities. (B) Average stride length. (C) Net knee moment over a gait cycle, normalized to body mass. (D) Average negative muscle moment during swing, normalized to body mass. (E) The moment applied by the device about the user’s knee over a gait cycle, normalized to body mass. (F) Percentage of the negative muscle impulse applied by the device during the swing period. (G) Average mechanical power removed. ***p < 0.001. Ψ and Θ indicate a significant difference between the given condition and normal walking or weighted walking, respectively (*p* < 0.05). Data presented as the mean ± S.E.M. Ave.: Average; Cont.: Contact; Int.: Initial; Mod.: Moderate; Term.: Terminal; Weight.: Weighted.

When comparing the kinematic and kinetic parameters of the five walking conditions, we found minimal changes in gait. We identified a statistically significant effect of assistance magnitude on peak hip flexion angle (*n* = 12 per condition; one-way repeated-measures ANOVA: *F*_*(2*.*248*,*24*.*730)*_ = 4.061, *p* = 0.026, *ηp*^2^ = 0.270). Our Šidák *post hoc* analysis revealed a slight increase (mean diff.: 0.7 ± 0.1 degrees) in peak hip flexion in the moderate assistance magnitude condition relative to that of normal walking (*p* < 0.001). Furthermore, we identified that assistance magnitude significantly affected peak ankle joint power (*F*_*(4*,*44)*_ = 3.209, *p* = 0.021, *ηp*^2^ = 0.226). Our *post hoc* analysis identified an increase in the peak ankle joint power of the high assistance condition compared to that of the moderate assistance condition (mean difference: 0.021 W·kg^-1^, *p* = 0.038). Additionally, we found a statistically significant effect of assistance magnitude on peak knee flexion angle (*F*_*(1*.*885*, *20*.*731)*_ = 4.152, *p* = 0.032, *ηp*^2^ = 0.274) and knee range of motion (*F*_*(1*.*791*,*19*.*697)*_ = 3.736, *p* = 0.046, *ηp*^2^ = 0.254); however, our follow-up *post hoc* analysis revealed no significant differences between conditions (all *p*s > 0.138). Minimal changes in joint kinematics and kinetics indicate that none of the walking conditions substantially impeded normal knee joint function (Fig 4A–4D) or the user’s natural gait (S3 Fig). The lack of effects of the exoskeleton on gait was further confirmed by a lack of changes in the participants’ spatial–temporal gait parameters, including stride length (Fig 4B), stride period, and ground contact time (all *F*s < 2.571; all *p*s > 0.096).

We found that there was a significant effect of assistance magnitude on the estimated average muscle contribution to the negative net knee moment applied during the swing period (*F*_(1.779,19.572)_ = 22.121, *p* < 0.001, *ηp*^2^ = 0.668; Fig 4D). Compared to that of normal walking, the low, moderate, and high assistance magnitudes reduced the average muscle contribution by 8.1 ± 1.8% (*p* = 0.012), 10.0 ± 1.5% (*p* = 0.003), and 15.2 ± 2.3% (*p* = 0.006), respectively. The weighted walking condition did not significantly alter the average muscle contribution relative to that of normal walking (*p* = 0.999).

The average moment profiles applied by the device about the user’s knee over a gait cycle under the three exoskeleton conditions are illustrated in Fig 4E. Although we attempted to change only the magnitude of assistance across conditions, differences in the shape of the assistance profiles also occurred, which changed the timing of the peak moment applied by the exoskeleton about the knee (F(1.152,12.670) = 72.020, p < 0.001, ηp2 = 0.868). Although the timing of the peak moment applied during the low magnitude condition (76.4 ± 1.4%) was significantly different from that of the moderate (89.6 ± 0.7%, p < 0.001) and high (88.4 ± 0.6%, p < 0.001) assistance magnitude conditions, the peak moment timings of the moderate and high assistance magnitude conditions were not significantly different (p = 0.076).

The low assistance magnitude profile more closely resembled an open-circuit loading condition (i.e., with the generator disconnected) than did the muscle-centric profile. At this low assistance magnitude, the force related to the mechanical system was the main contributor to the cable force. Therefore, because the force associated with the mechanical system is uncontrolled, unlike the force related to the electrical system, the loading profile took the shape of the open-circuit condition demonstrated in [30]. This result highlights the importance of reducing the mechanical system’s cable force contribution during a device’s design, especially if low-magnitude assistance is needed. It also highlights why we could not evaluate an assistance level between 0% and 10%.

The actual assistance magnitudes applied during the low, moderate, and high assistance conditions were 10.1 ± 1.0, 14.9 ± 1.2, and 20.1 ± 1.5% of the estimated muscle contribution to the rotation impulse about the knee, respectively (Fig 4F). We found that the assistance magnitudes applied were not significantly different from the desired assistance levels (i.e., low: 10%, moderate: 15%, and high: 20%; one-sample *t*-test, all *t*s < 0.135, *p* > 0.895) but were significantly different between conditions (*F*_*(2*,*22)*_ = 125.950, *p* < 0.001, *ηp*^*2*^ = 0.920; Šidák *post hoc* analysis: all *p*s < 0.001). This finding suggests that the impulse controller effectively regulated the desired impulse applied by the exoskeleton. We also found that the three assistance magnitude conditions removed significantly different amounts of mechanical power (*F*_*(2*,*22)*_ = 84.771, *p* < 0.001, *ηp*^*2*^ = 0.885; Šidák *post hoc* analysis: all *p*s < 0.001). The amount of mechanical power removed increased with increasing assistance magnitude (Fig 4G). The low, moderate, and high assistance magnitude conditions removed 1.1 ± 0.1 W, 2.1 ± 0.2 W, and 3.0 ± 0.2 W, respectively.

### Electrical power production

**Fig 5:**
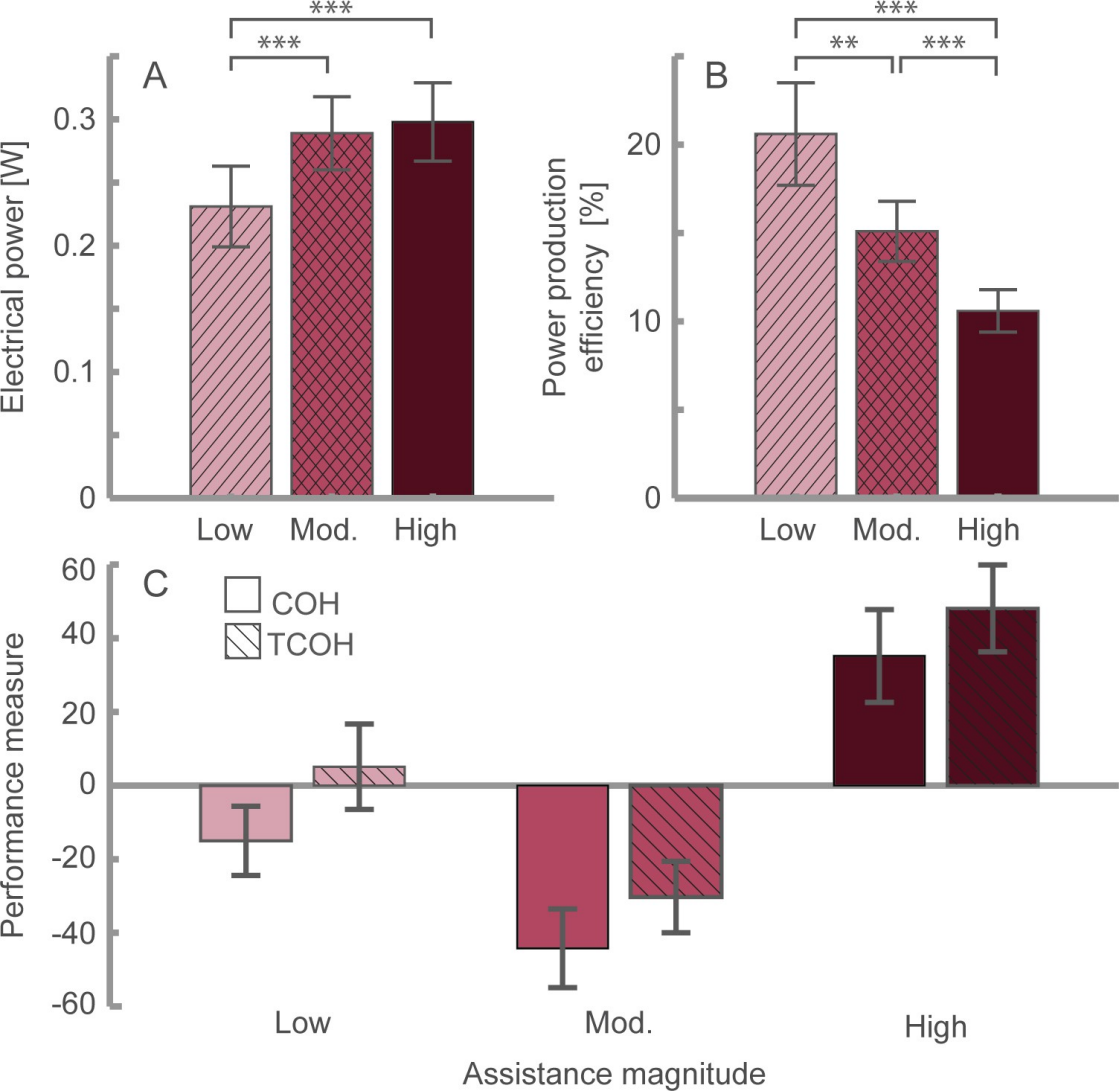
Electrical power production and device performance measures. The (A) electrical power production and (B) device electrical power production efficiency under the low, moderate, and high assistance conditions. (C) The device’s Cost of Harvest (COH) and Total Cost of Harvest (TCOH; hatched) under the low, moderate, and high assistance conditions. **p < 0.01, ***p < 0.001. Data presented as the mean ± S.E.M. Mod.: Moderate.

We identified that the assistance magnitude significantly altered the amount of electrical power produced by the exoskeleton (*F*_(*2*,*22*)_ = 11.721, *p* < 0.001, *ηp*^*2*^ = 0.516; Fig 5A). Our *post hoc* comparisons revealed that the device produced significantly less electrical power during the low assistance condition than during the moderate (*p* = 0.004) and high (*p* = 0.013) assistance conditions. However, we determined that the moderate and high assistance conditions did not significantly differ in electrical power production (*p* = 0.851). The device produced 0.23 ± 0.03 W, 0.29 ± 0.03 W, and 0.30 ± 0.03 W of electrical power under the low, moderate, and high assistance conditions, respectively.

Furthermore, we determined that the assistance magnitude significantly altered the electrical power production efficiency of our device (*F*_*(1*.*149*,*12*.*643)*_ = 18.811, *p* < 0.001, *ηp*^*2*^ = 0.631; Fig 5B). The efficiency of electrical power production during low, moderate, and high assistance was 20.6 ± 2.9%, 15.1 ± 1.7%, and 10.6 ± 1.2%, respectively. Our device’s electrical power production efficiency decreased with increasing assistance magnitude because, in order for the device to apply higher cable loads, the control system increased the applied electromagnetic force by decreasing the electrical resistance of the electrical power circuit [30]. As the power circuit’s electrical resistance approached the generator’s internal resistance, greater thermoelectric losses occurred within the generator. Therefore, the additional mechanical power removed from the body under the high assistance condition was lost as heat within the generator and not converted into electricity. This loss resulted in the observed plateau in electrical power production between the moderate and high assistance conditions (Fig 5A).

To quantify the metabolic effort required to generate electrical power, we calculated the energy harvesting performance measures COH and TCOH for each exoskeleton condition (Fig 5C). The COH under the low, moderate, and high assistance magnitude conditions was -15.0 ± 9.4, -44.2 ± 10.7, and 35.2 ± 12.6, respectively. The TCOH under the low, moderate, and high assistance conditions was 5.1 ± 11.6, -30.3 ± 9.7, and 48.1 ± 11.8, respectively. The negative TCOH indicates that a user would benefit from a decreased metabolic cost of walking with our device, regardless of the need for portable power.

## Discussion

We examined the energetic and biomechanical effects of walking with an energy-removing exoskeleton that was designed to assist the hamstrings in producing force while applying different assistance magnitudes. Under the moderate assistance condition, the device reduced the metabolic cost of walking below that of normal walking by 3.4% while producing 0.29 W of electrical power. This reduction in the energetic cost of walking was associated with an 8.9% decrease in hamstring activity. Although previous studies have shown a quadratic relationship between the metabolic cost of walking with active and passive exoskeletons and the device’s assistance magnitude [7,10], no such relationship was observed under the present experimental conditions (*R*^*2*^ = 0.018; *p* = 0.672). We found that the metabolic cost of walking slowly decreased below that of normal walking as the assistance magnitude increased from zero to a moderate level; however, as the assistance magnitude increased above the moderate level, the metabolic cost dramatically increased to exceed that of weighted walking. In addition to the substantial increase in metabolic cost between the moderate and high assistance conditions (9.6 ± 2.1%), the hamstring activity of the high assistance condition was also significantly greater than that of the moderate condition (11.8 ± 2.4%).

This transition from assisting to hindering the user may have resulted from the device disrupting natural gait and muscle mechanisms. Some soft tissues such as ligaments store and return energy [45,46] during walking. At higher assistance magnitudes, the device could disrupt these energy return mechanisms, resulting in an energetic penalty caused by the body compensating through concentric contractions. Because concentric contractions are much more metabolically costly than eccentric muscle contractions [47], slight increases in concentric muscle activity could result in a drastic increase in the metabolic cost of walking. Although we did not observe an increase in overall quadriceps muscle activity (0–100% gait cycle) for the high assistance condition, the increased metabolic cost of walking observed during this walking activity could be due to the increased quadricep activity observed during late swing (75–100% gait cycle). This increase may have resulted from the body counteracting the load applied by the device during this period to ensure adequate knee extension or provide knee stability.

The shallow metabolic assistance slope observed with low to moderate assistance magnitudes may be due to eccentric contractions being relatively metabolically efficient compared to the energy required to assist concentric contractions [47]. Therefore, their assistance would only lead to a relatively small metabolic reduction compared to an increase related to concentric muscle contractions. This observation indicates that there is a threshold for assisting eccentric contractions; crossing this threshold by extracting too much energy from the body could lead to a substantial metabolic penalty. However, further research is needed to identify the assistance magnitude of this threshold. Furthermore, the magnitude is most likely user-specific—suggesting that future adaptive control systems that identify user-specific parameters may yield greater metabolic cost reductions than non-adaptive systems.

Active exoskeletons may not exhibit this sudden increase in metabolic cost because, if these devices were to disrupt their user’s natural gait, the body could perform metabolically efficient eccentric contractions to counterbalance the device. Because eccentric contractions are more efficient than concentric contractions, performing eccentric contractions to counteract a device may be less metabolically costly than counteracting a device through concentric contractions. If passive devices were to disrupt their user’s natural gait mechanisms when collecting and transferring energy, the isometric and concentric muscle assistance they provide could mask these adverse metabolic effects.

Furthermore, the high assistance magnitude condition in the present study may have caused a slight but undetectable gait abnormality resembling stiff knee gait, which is often associated with a greater metabolic cost of walking [48]. For example, if the device caused a slight decrease in knee flexion (i.e., by increasing the swinging limb length), this would result in the swinging limb having a larger moment of inertia [49,50]. This larger moment of inertia would require the user’s muscles to generate larger hip moments, resulting in increased metabolic expenditure. Additionally, the user may adopt compensatory mechanisms such as a slight increase in hip circumduction, hip hiking, lateral trunk lean, or contralateral vaulting to overcome the increased swinging limb length and reduce the risk of the toe scuffing the walking surface [51].

To investigate why the reduction in metabolic cost depends on assistance magnitude, we compared the effects of different assistance magnitudes on gait kinematics and kinetics and muscle activity. Interestingly, we found that none of the assistance magnitudes substantially altered gait kinematics and kinetics relative to those of normal or weighted walking. Similarly, none of the assistance magnitudes significantly altered the overall quadriceps activity. This finding may indicate that the exoskeleton assistance conditions, specifically the high assistance condition, increased the activity of muscles crossing the ankle or hip. Therefore, further experiments are required to clarify the cause of the relationship between metabolic cost and assistance magnitude observed in the present study.

Removing energy during the swing period using our device may indirectly affect the user during the stance period. For instance, the moderate assistance condition appeared to decrease hamstring activity during early stance (i.e., 0–33% gait cycle, Fig 3A) compared to normal walking. During early stance, the already diminished activity of the hamstrings continues to wane, but it still provides knee joint stability during initial contact (0–2% gait cycle) to prevent hyperextension. Therefore, removing energy during swing may help prepare the lower limb for initial contact and load acceptance.

Despite the moderate assistance condition appearing to decrease the average hamstring activity during early stance relative to normal walking (Fig 3A), hamstring activity did not significantly change (*p* = 0.369). However, a decrease in hamstring activity during early stance would not be surprising because the moderate assistance condition significantly reduced hamstring activity during late stance, resulting in less activity to wane during early stance. Because of the suspected limited force production by the hamstrings during early stance, it is unclear how decreased activity during early stance would reduce the metabolic cost of walking. Nevertheless, removing energy during the swing phase may have positive indirect effects during the stance phase.

The present results align with those of our previous study [8], which evaluated the effects of assistance timing. Comparing the muscle-centric profile condition from our previous study to the muscle-centric profile that applied a moderate level of assistance in the present study, we found that both conditions resulted in similar biomechanical and energetic effects. For instance, the previous study demonstrated that the muscle-centric profile reduced the metabolic cost of walking and hamstring activity relative to that of normal walking by 2.5 ± 0.8% and 11.2 ± 3.8%, respectively, whereas the moderate assistance condition in the present study reduced the metabolic cost of walking and the hamstring activity relative to that of normal walking by 3.4 ± 1.0% and 8.9 ± 2.3%, respectively. Under these two conditions, the exoskeleton removed a similar amount of mechanical power (previously: 2.0 ± 0.1 W; presently: 2.1 ± 0.2 W), applied a similar percentage of the muscles’ angular impulses (previously: 17.4 ± 1.5%; presently: 14.9 ± 1.1%), and applied a peak knee moment at approximately the same percentage of the gait cycle (previously: 90.7 ± 0.2%; presently: 89.6 ± 0.7%). The similarity in these two studies’ results demonstrates the robustness of the exoskeleton’s metabolic effects under the presented experimental conditions.

The electrical power production of our device can be increased by increasing its power conversion efficiency. This can be accomplished by designing a generator with characteristics better suited for this application. A generator with more desirable characteristics would decrease thermoelectric losses within the generator. Furthermore, the device’s power conversion efficiency could be increased by implementing a power electronics module consisting of a digitally controlled average current mode boost converter and an average current mode buck converter [52]. However, further research is needed as generators operate most efficiently at high velocities (e.g., >1000 rpm), whereas gait is associated with slower joint velocities (e.g., knee: ∼20 rpm) [53]. Nevertheless, our device’s negative TCOH and COH indicates that a user would benefit from a decreased metabolic cost of walking, regardless of the need for portable power.

## Limitations

The present work provides evidence that assistance magnitude is a critical parameter when providing assistance with an energy-removing exoskeleton or biomechanical energy harvester while walking; however, potential limitations should be noted. One limitation is that we only tested our device on male participants. We focused on one sex to decrease the between-subject variability caused by sex differences in body mass distribution [54]. High between-subject variability in body mass distribution would be problematic because we scaled the assistance profile to the user’s body mass and because we expected the device to yield only a small amount of metabolic assistance. Additional experiments are needed to determine the nature and extent of the exoskeleton’s assistance in females; however, we do not believe that the present findings would differ as a function of sex.

Another limitation of the present work is that we conducted the device assessments on a level treadmill with a fixed walking speed. This artificial walking environment enabled a controlled comparison between multiple walking conditions, but it does not allow for real-world device testing. In addition to limiting the generalizability of the present findings, the walking environment may have constrained the participants’ ability to negotiate the load applied by the exoskeleton. For example, the load may have naturally caused users to slow down, but the fixed belt speed of the treadmill prevented them from doing so. However, such effects of the device would most likely have manifested in spatial and temporal gait changes, so would not have gone unnoticed.

## Conclusions

The present study demonstrated that the assistance magnitude of an energy-removing exoskeleton designed to assist the hamstrings in force production can significantly affect the metabolic cost, muscle activity, and biomechanics of walking. Under the moderate assistance condition, the exoskeleton reduced the metabolic cost of walking below that of normal walking by 3.4% while producing 0.29 W of electrical power. Furthermore, this reduction in metabolic cost was associated with an 8.9% decrease in hamstring activity compared to that of normal walking. Our results also demonstrate the existence of an assistance threshold that, when crossed, significantly increases the metabolic cost of walking and muscle activity. These results could aid in the future design of adaptive control systems that identify user-specific control parameters associated with minimum energy expenditure.

While the present work has identified assistance magnitude as a critical factor that affects the metabolic cost of walking while removing energy, further work is required to determine the conditions under which assistance magnitude is most important. For instance, metabolic assistance will likely differ according to walking speed and the slope of the walking surface. Investigations comparing multiple energy-removal parameters, such as assistance timing and magnitude, will clarify the most effective strategies for providing walking assistance.

## Supporting information

S1 Fig Activity of the hamstring muscle group and individual muscles. Muscle activity profiles of the five walking conditions (normal walking: Solid grey; weighted walking: Dashed dark grey; low assistance magnitude: Solid light red; moderate assistance magnitude: Dotted red; and high assistance magnitude: Dash-dot dark red) for the hamstring muscle group (A) and the biceps femoris (C) and semitendinosus (E) muscles. Average hamstring muscle group (B), biceps femoris (D), and semitendinosus (F) muscle activity for an entire gait cycle. *p < 0.05, **p < 0.01. Data presented as the mean ± S.E.M. Cont.: Contact; Int.: Initial; Mod.: Moderate; Term.: Terminal; Weight.: Weighted.
(TIF)

S2 Fig Activity of the quadriceps muscle group and individual muscles. Muscle activity profiles of the five walking conditions (normal walking: Solid grey; weighted walking: Dashed dark grey; low assistance magnitude: Solid light red; moderate assistance magnitude: Dotted red; and high assistance magnitude: Dash-dot dark red) for the quadriceps muscle group (A) and the rectus femoris (C), vastus lateralis (E), and vastus medialis (G) muscles. Average quadriceps group (B), rectus femoris (D), vastus lateralis (F), and vastus medialis (H) muscle activity for an entire gait cycle. *p < 0.05, **p < 0.01, ***p < 0.001. Data presented as mean ± S.E.M. Cont.: Contact; Int.: Initial; Mod.: Moderate; Term.: Terminal; Weight.: Weighted.
(TIF)

S3 Fig Joint kinematics and kinetics averaged over participants for the five walking conditions. Average sagittal plane net joint angles (A–C), moments (D–F), and powers (G–I) for the right ankle (A, D, G), knee (B, E, H), and hip (C, F, I) over a gait cycle for the following five walking activities: Normal walking (solid grey); weighted walking (dashed dark grey); low assistance magnitude (solid light red); moderate assistance magnitude (dotted red); and high assistance magnitude (dash-dot dark red).
(TIF)

S1 Data Experimental metadata and statistical findings. Data file including metadata and results of statistical analyses.
(ZIP)

S2 Data Experimental data. Data file including de-identified biomechanical and physiological data.
(ZIP)
