# Supplementary figures and images for: A transition point: Assistance magnitude is a critical parameter when providing assistance during walking with an energy-removing exoskeleton or biomechanical energy harvester
Source: PLoS One. 2023 Aug 10;18(8):e0289811. doi: 10.1371/journal.pone.0289811 (PMC10414649; doi:10.1371/journal.pone.0289811)

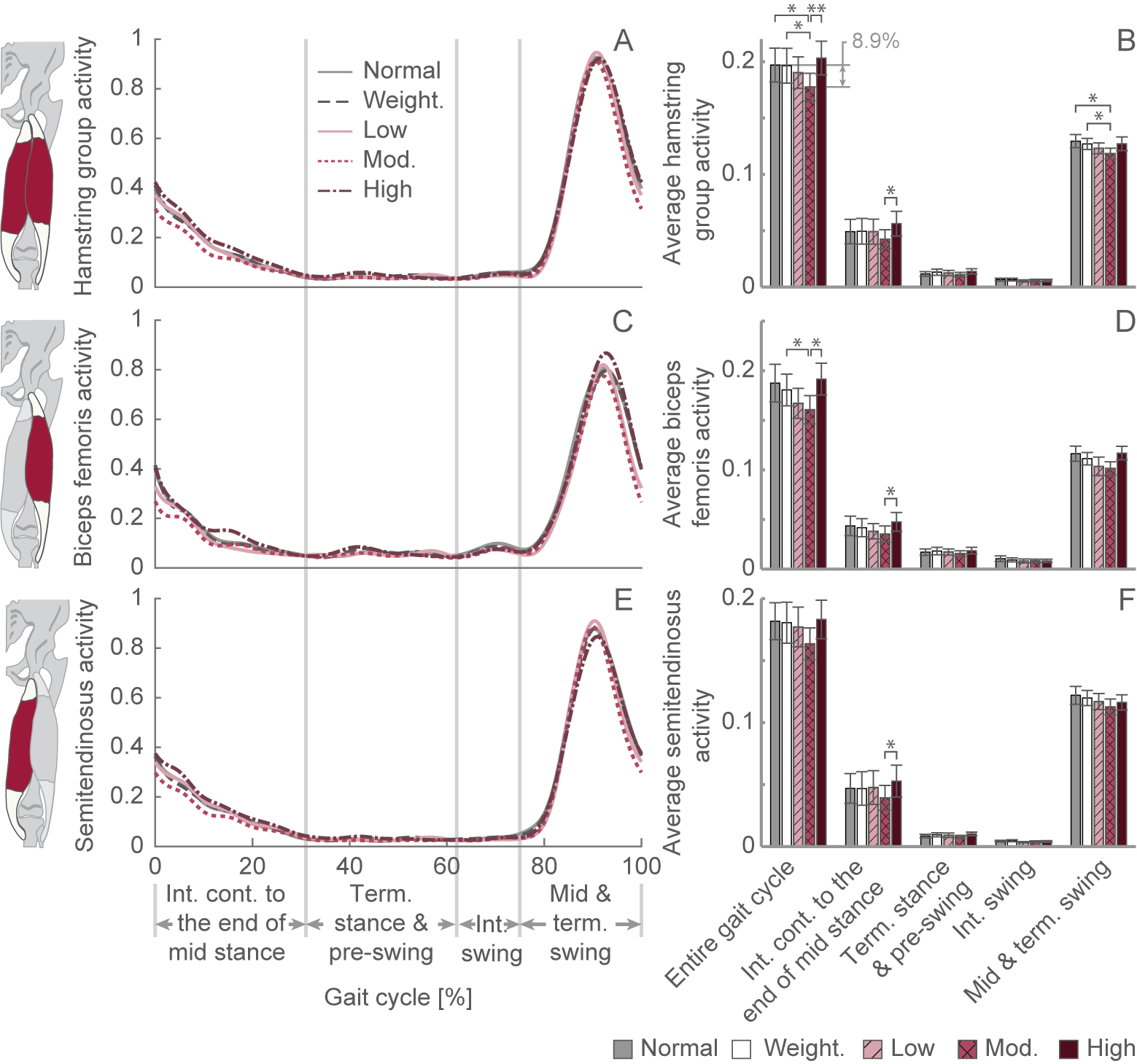

Supplement: S1 Fig — Muscle activity profiles of the five walking conditions (normal walking: Solid grey; weighted walking: Dashed dark grey; low assistance magnitude: Solid light red; moderate assistance magnitude: Dotted red; and high assistance magnitude: Dash-dot dark red) for the hamstring muscle group (A) and the biceps femoris (C) and semitendinosus (E) muscles. Average hamstring muscle group (B), biceps femoris (D), and semitendinosus (F) muscle activity for an entire gait cycle. *p < 0.05, **p < 0.01. Data presented as the mean ± S.E.M. Cont.: Contact; Int.: Initial; Mod.: Moderate; Term.: Terminal; Weight.: Weighted. (TIF) [file pone.0289811.s001.tif]

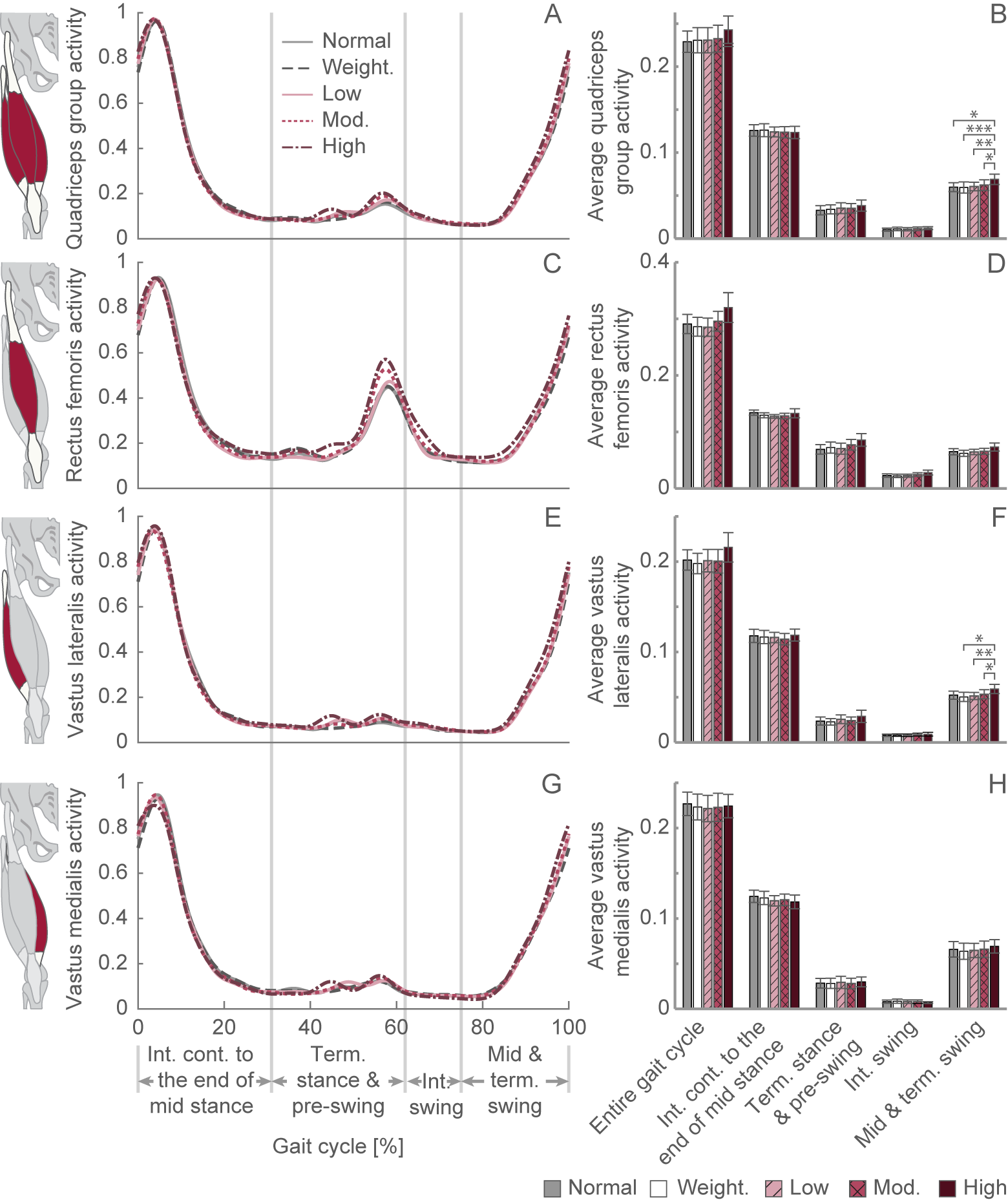

Supplement: S2 Fig — Muscle activity profiles of the five walking conditions (normal walking: Solid grey; weighted walking: Dashed dark grey; low assistance magnitude: Solid light red; moderate assistance magnitude: Dotted red; and high assistance magnitude: Dash-dot dark red) for the quadriceps muscle group (A) and the rectus femoris (C), vastus lateralis (E), and vastus medialis (G) muscles. Average quadriceps group (B), rectus femoris (D), vastus lateralis (F), and vastus medialis (H) muscle activity for an entire gait cycle. *p < 0.05, **p < 0.01, ***p < 0.001. Data presented as mean ± S.E.M. Cont.: Contact; Int.: Initial; Mod.: Moderate; Term.: Terminal; Weight.: Weighted. (TIF) [file pone.0289811.s002.tif]

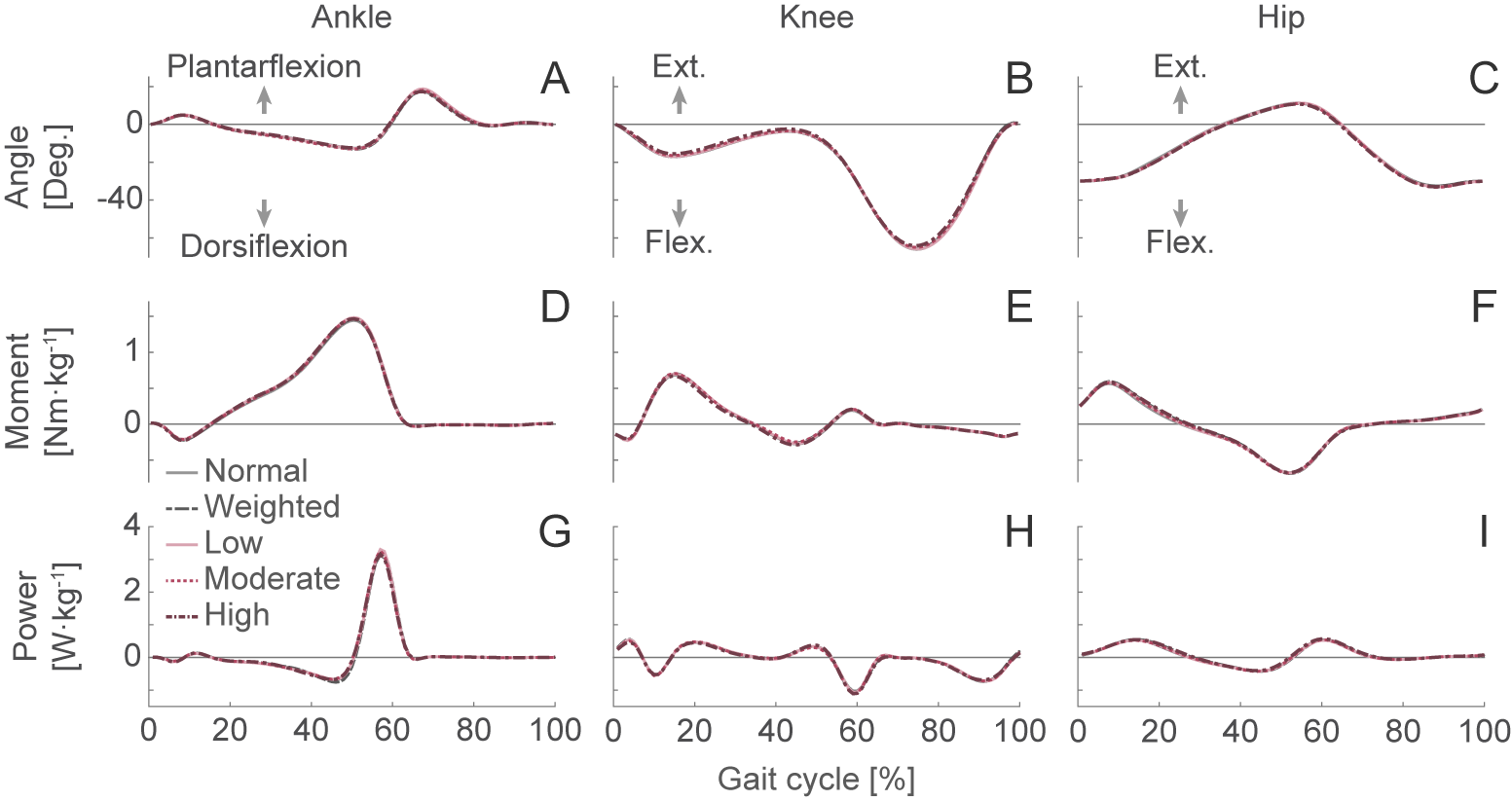

Supplement: S3 Fig — Average sagittal plane net joint angles (A–C), moments (D–F), and powers (G–I) for the right ankle (A, D, G), knee (B, E, H), and hip (C, F, I) over a gait cycle for the following five walking activities: Normal walking (solid grey); weighted walking (dashed dark grey); low assistance magnitude (solid light red); moderate assistance magnitude (dotted red); and high assistance magnitude (dash-dot dark red). (TIF) [file pone.0289811.s003.tif]
